# Kandinsky Clerambault syndrome in a patient with treatment-resistant schizophrenia - elements of psychiatric semiology

**DOI:** 10.1192/j.eurpsy.2023.2070

**Published:** 2023-07-19

**Authors:** L. A. Ignat, R. Tipa, P. Tirlea, A. Iacob

**Affiliations:** 1 “Prof. Dr. Al. Obregia” Psychiatry Clinical Hospital, Bucharest, Romania

## Abstract

**Introduction:**

Nothing is taken more for granted than the feeling that we are in control of our own thoughts and actions. For those who experience thoughts insertion or delusion of control, known as first rank symptoms of schizophrenia, it becomes a luxury to rule their own thoughts. Kandinsky-Clerambault syndrome is characterized by pseudo-hallucinations, delusions of control, telepathy, thought broadcasting and thought insertion by an external force. The patient’s thoughts, emotions, perceptions or actions are under the control of a different agent, or sometimes he believes that the operator of control is inside his body.

**Objectives:**

Presentation of a clinical case of Kandinsky-Clerambault syndrome in a patient with treatment-resistant schizophrenia.

**Methods:**

Case report

**Results:**

We present the case of a 38-year-old man, diagnosed with paranoid schizophrenia since 2014, who has followed several therapeutic plans, starting with Haloperidol followed by Risperidone, Paliperidone, Quetiapine, Clozapine. Since 2016 he had been under treatment with Xeplion injectable 150mg/month ambulatory, but the psychotic features never fully remitted. 10 days before admission he had last administration of Xeplion LAI. The patient reports the loss of control over his mental life and describes the triple automatism: ideo-verbal, sensory and motor. *“There are some people in my body who control me, they move my limbs. I feel like my body is not mine.*” He describes imperative and commentative auditory pseudo-hallucinations. The patient speaks intermittently in the 3rd person about himself, has circumstantial discourse, with elements of tangentiality, ideo-verbal and conceptual disorganization. He presents delusions of control, persecution and prejudice. The treatment received during admission – Riseridone 5ml/day, Amisulpride 600mg/day, Orfiril Long 1000mg/day.

**Conclusions:**

After 14 days of hospitalization, the patient is discharged in an improved state, without mentioning spontaneously the delusional ideation. He affirms the intermittent presence of auditory pseudo-hallucinations, but with low intensity compared to the moment of admission. It is ironic how the loss of the self, along with the insertion of thoughts and auditory pseudo-hallucinations create a patient’s own reality, which at the same time is experienced as coming from the outside. From a phenomenological point of view, thought insertion is explained as an autoimmune disease. Thoughts are produced by our mind, but because we have lost the meaning of control and belonging to ourselves, those thoughts are attacked as foreign.

**Disclosure of Interest:**

None Declared

